# OCTN1: A Widely Studied but Still Enigmatic Organic Cation Transporter Linked to Human Pathology and Drug Interactions

**DOI:** 10.3390/ijms23020914

**Published:** 2022-01-14

**Authors:** Lorena Pochini, Michele Galluccio, Mariafrancesca Scalise, Lara Console, Gilda Pappacoda, Cesare Indiveri

**Affiliations:** 1 Unit of Biochemistry, Molecular Biotechnology and Molecular Biology, Department of Biology, Ecology and Earth Sciences (DiBEST), University of Calabria, Via P. Bucci 4c, Arcavacata di Rende, 87036 Cosenza, Italy; lorena.pochini@unical.it (L.P.); michele.galluccio@unical.it (M.G.); mariafrancesca.scalise@unical.it (M.S.); lara.console@unical.it (L.C.); gilda.pappacoda@gmail.com (G.P.); 2 Institute of Biomembranes, Bioenergetics and Molecular Biotechnology (IBIOM), National Research Council—CNR, Via Amendola 122/O, 70126 Bari, Italy

**Keywords:** acetylcholine, ergothioneine, carnitine, health, inflammation, oxidative stress, pharmacology, cancer

## Abstract

The Novel Organic Cation Transporter, OCTN1, is the first member of the OCTN subfamily; it belongs to the wider Solute Carrier family SLC22, which counts many members including cation and anion organic transporters. The tertiary structure has not been resolved for any cation organic transporter. The functional role of OCNT1 is still not well assessed despite the many functional studies so far conducted. The lack of a definitive identification of OCTN1 function can be attributed to the different experimental systems and methodologies adopted for studying each of the proposed ligands. Apart from the contradictory data, the international scientific community agrees on a role of OCTN1 in protecting cells and tissues from oxidative and/or inflammatory damage. Moreover, the involvement of this transporter in drug interactions and delivery has been well clarified, even though the exact profile of the transported/interacting molecules is still somehow confusing. Therefore, OCTN1 continues to be a hot topic in terms of its functional role and structure. This review focuses on the most recent advances on OCTN1 in terms of functional aspects, physiological roles, substrate specificity, drug interactions, tissue expression, and relationships with pathology.

## 1. Introduction

**Figure 1:**
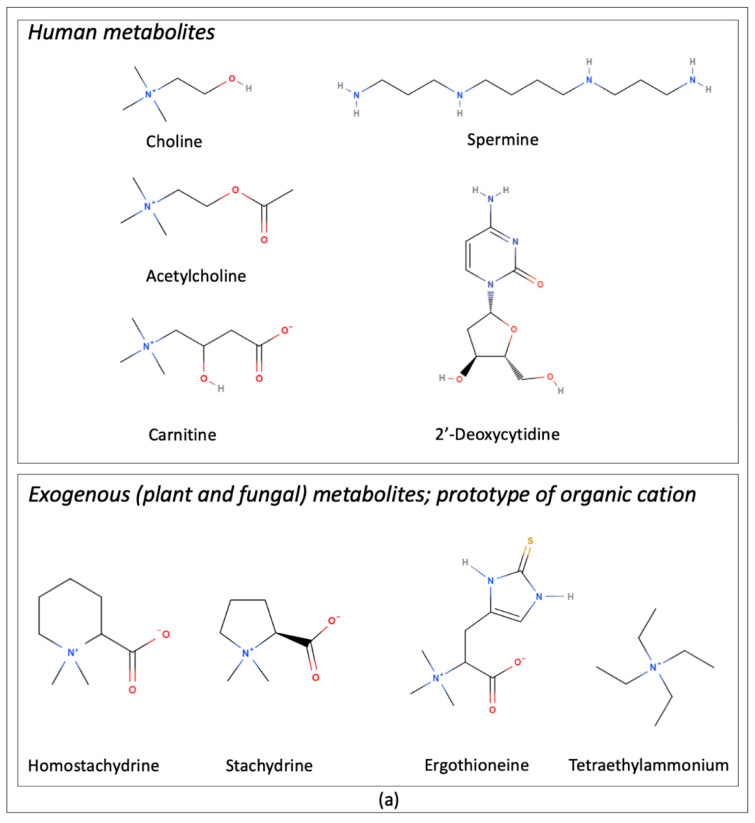

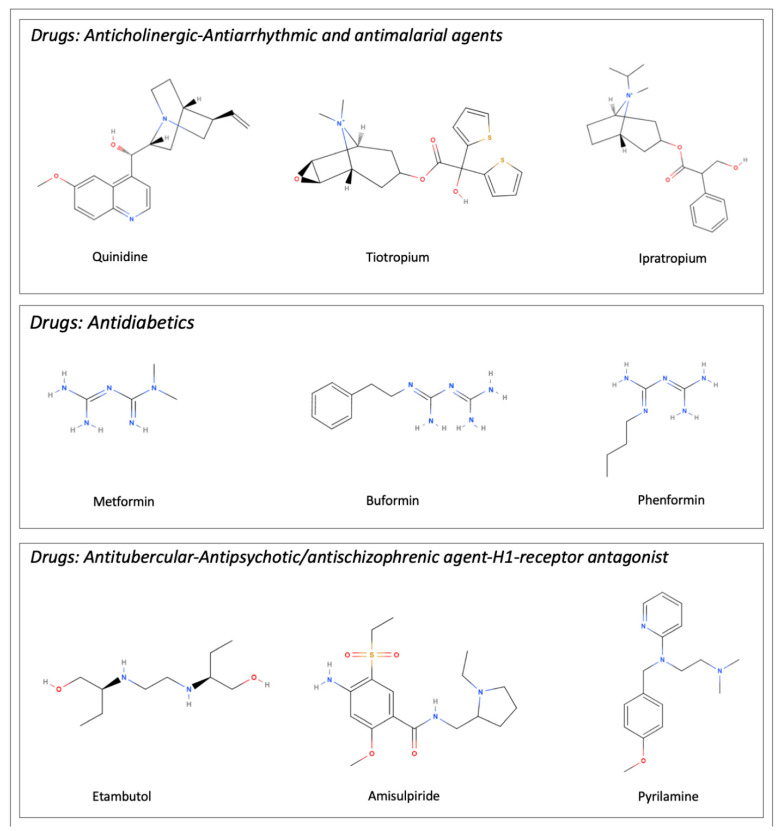

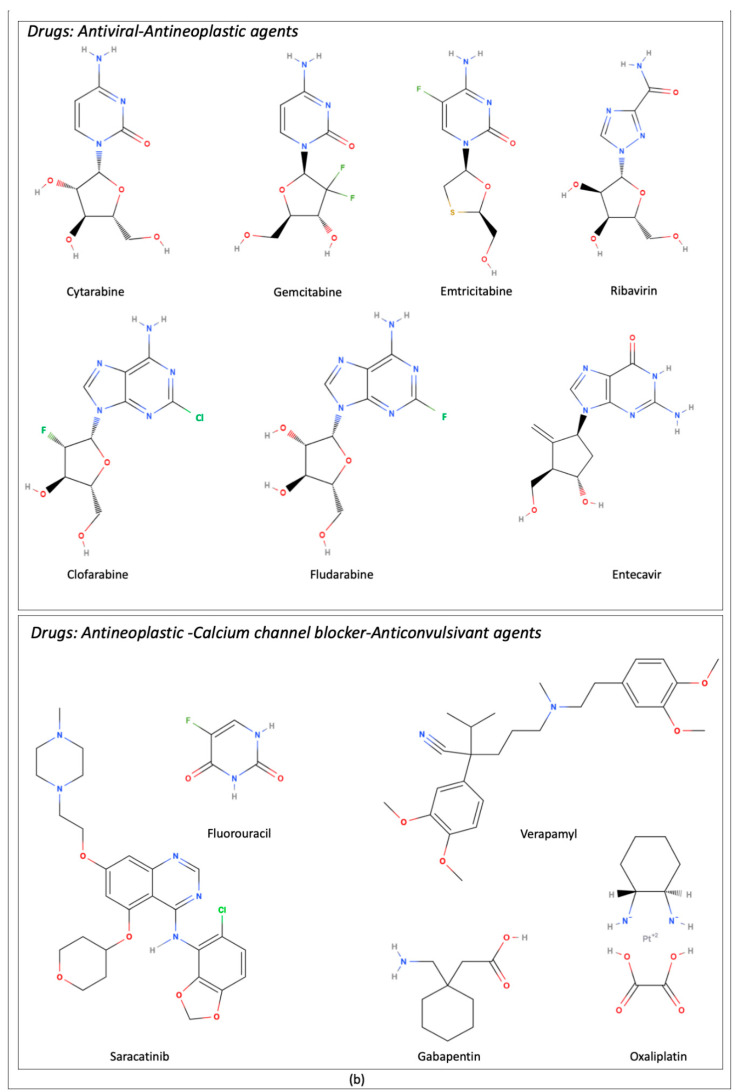
Image of the structural formula of molecules for which transport mediated by the Novel Organic Cation Transporter 1, OCTN1 has been demonstrated. Nitrogen atoms are colored in blue, Oxygen atoms in red, Hydrogen atoms in gray, Halogen atoms in green. (**a**) Human and Exogenous metabolites. (**b**) Drugs, organized in different panels on the basis of structure similarities.

OCTN1 (SLC22A4) is the first member of the human Novel Organic Cation Transporters small subfamily, which is part of the larger SLC22 family. These transporters appeared in evolution with vertebrates, thus being particularly interesting also for human metabolism [1]. Besides terrestrial animals closer to humans, OCTN1 is also present in aquatic species such as *Danio rerio* (zebra fish) [2] and rainbow trout [3]. In the latter species, the capacity of OCTN1 in mediating ergothioneine transport has been tested, even though no information on a possible physiological function of ergothioneine in salmonids is available [3]. In spite of its late appearance in evolution, OCTN1 shows 25% sequence identity with the memory suppressor protein from *Drosophyla melanogaster* CG7442. Interestingly, in analogy with some described functional features of OCTN1, CG7442 (dmSLC22A) was found to have choline and acetylcholine as the preferred substrates (but not carnitine), performing the role of removing neurotransmitters from the synapse [4]. Furthermore, the protein sequence of T08B1.1 of the invertebrate *Caenorhabditis elegans* showed OCTN1 as the top matched human sequence. By a clustalX analysis, an identity of 17.6% and a similarity of 42.7% have been calculated. T08B1.1 has been described as responsible for L-carnitine uptake, promoting oxidative stress recovery and increasing lifespan [5]. Despite a close proximity between the two human OCTN members, in terms of sequence identity (76%) [6] and gene localization (IBD5 locus of chromosome 5q31), the role of OCTN1 in human metabolism is still uncertain. The most well described substrates of OCTN1 are some organic cations and zwitterions; however, carnitine, the well assessed zwitterion substrate of the Novel Organic Cation Transporter 2, OCTN2, is transported by OCTN1 at a very low efficiency. Accordingly, OCTN1 could not compensate for the genetic defect responsible for the primary carnitine deficiency, an autosomal recessive disorder caused by mutations in the *OCTN2* gene [7]. Evidence of *OCTN1* genetic variants linked to carnitine deficiency has not been described, confirming that it is not relevant for carnitine transport in vivo [8]. Concerning the organic cation group of molecules, the first identified OCTN1 substrate was tetraethylammonium (TEA), which allowed one to functionally discriminate this transporter from OCTN2. However, TEA cannot be classified among the physiological substrates as it is an exogenous prototype organic cation [9]. Many efforts were made to search for physiological substrates. The mushroom metabolite ergothioneine was first identified as a substrate of OCTN1 [10] (see Figure 1). 

Soon after, acetylcholine was identified as a substrate among other organic cations [7,11] such as choline, spermine, etc. (see Table 1 and Figure 1a) [7,12,13,14]. 

Besides the described zwitterion ergothioneine and the organic cation acetylcholine, many drugs have been proposed to be substrates or interactors of OCTN1 (see Table 2 and Figure 1b) [6,7,15]. Indeed, *OCTN1* as a member of the SLC22 family could be classified in the so called ADME genes, which are transporters and enzymes conventionally viewed as central to the absorption (A), distribution (D), metabolism (M), and elimination (E) of drugs [19,20]. Thus, interest in OCTN1 was also focused on its involvement in drug interaction and delivery [21,22]. On the basis of the many different molecules described as substrates or inhibitors of OCTN1, a main question arose on whether the transporter has a strict specificity for a few molecules or a wide specificity for different groups of molecules. An unequivocal response to this question is still lacking since the three-dimensional structure of OCTN1 is still unsolved and, hence, the molecular determinants for substrate interaction are not clearly assessed.

Concerning tissue/organ/cell and subcellular localization, data collected to date, referring both to mRNA or protein expression, indicate that OCTN1 is a ubiquitous transporter [7]. After more than 20 years of research, the gathered information outlines an unclear picture of this transporter and of its role in human physiology or pathology, allowing mostly speculative hypotheses. In this review we will summarize the most recent achievements on OCTN1 and on perspectives of defining its role that still lies at the border between a physiological and xenobiotic compound transporter. 

## 2. Tissue Expression/Localization of OCTN1

Based on the differences existing among species in terms of protein localization and/or expression levels, transferring information from animal models to humans is no longer considered a proper practice [7]. In this context, protein abundance data of 19 transporters in the kidney cortex across five species (human, monkey, dog, rat, and mouse) has been investigated [33]. The authors of this work found that the relative abundance of kidney OCTN1, measured by LC-MS/MS, revealed a higher or similar expression in human with respect to monkey. In other species, OCTN1 was “not classified” (NC). 

Knowledge of the tissue and level of expression both in physiological (see following Section 2.1) and pathological conditions (see Section 6.1) of a transporter like OCTN1 is twice as important since it could give some insights to the hot topic of understanding the physiological role of the transporter and link specific (transported) drugs to human diseases [34]. 

### 2.1. OCTN1 Expression in Relationships with Physiological Roles

Many reviews over the years have reported updates to the OCTN1 expression/localization in physiological conditions [6,7,19,35,36,37]. Very recently, different expression levels have been quantified in 56 different mammalian cell lines [36] as well as in 59 tissues [38]. Authors found OCTN1 mRNA and/or protein widely expressed in all the analyzed tissues, indicating that OCTN1 is a ubiquitous transporter. A higher expression level has been found in only some of these tissues/cells. 

Protein localization in immune cells together with the gene chromosomal localization, strongly suggest a role for OCTN1 in the immune system and in the inflammatory process [7]. Indeed, it has been known for many years that the presence of a combined two-allele haplotype in the coding region of *hOCTN1* is associated with an increased risk for chronic inflammatory diseases such as Crohn’s disease and ulcerative colitis [39]. More recently, to confirm a role in immunity, regions of the genome of sheep have been identified that were most likely associated with resistance to gastrointestinal nematode (GIN) infections [40] and *OCTN1* was among the genes associated with this resistance. 

Emphasis has been given to OCTN1 intestinal and kidney expression due to the role played by this SLC in pharmacokinetics and pharmacodynamics. OCTN1 has a commonly accepted apical localization in kidney. An apical intestinal localization has been suggested as well [41] and PDZ-containing kidney protein (PDZK) interaction would corroborate this localization [42]. On the basis of relative abundance data, some authors revealed that region specific expression was highest in the proximal jejunum and lowest in the colon [43]. However, more detailed data would be necessary to unequivocally assess the intestinal OCTN1 sub-localization. Subcellular localizations have also been revealed in other epithelia like the tracheal and bronchial alveolar epithelia [7,9]. This sub-cellular localization suggests a concerted action of OCTN1 with other transporters, which indeed share substrate specificity and are differentially located at the apical or the basolateral membranes. The transporters cooperate in moving substrates in one way, for example, in absorption processes (intestine), or in an opposite way, i.e., excretion processes (kidney). 

OCTN1 is expressed in the context of the nervous system as well [44], but, interestingly, controversial data are reported concerning its expression at the level of the nervous system’s check-point represented by the blood–brain barrier [7,21,22]. Indeed, quantification has been done in Brain Microvascular Endothelial Cells (BMECs), which are a central element of the microvasculature that forms the blood–brain barrier. Quantification has been performed from the occipital and the parietal regions of the brain. Authors found that abundance of OCTN1 was below the lower limit of quantification, which is 0.35 fmol-on-column [45]. This corroborates the hypothesis that other transporters share OCTN1 substrate specificity in order to ensure substrate entry into the nervous system. OCTN1 is expressed in the brain in neural stem cells, neurons, and in microglia, the “macrophages” of the brain [46], at much higher levels in neural stem cells compared with other Organic Cation Transporters, OCTs. 

On the basis of OCTN1 expression in tissues forming a metabolic axis (gut, brain, kidney), very recently, a role for OCTN1 in the context of the Remote Sensing and Signaling Theory (RSST) has been suggested [19,20] as part of a responsive/adaptive system allowing the inter/intra-organ (gut–brain, gut–kidney axis) and inter-organismal communication. Clarifying the OCTN1 role in intra-organ, inter-organ, and inter-organismal communication and in general in the human physio/pathology is under discussion. 

Expression in the placenta has particular importance. In this district OCTN1 could be a potential route for drug delivery to fetus. However, the OCTN1 abundance was not quantifiable in the placenta by quantitative targeted proteomics using liquid chromatography tandem mass spectrometry [47]. 

Expression of OCTN1 in the mammary gland has been investigated and found to vary with the lactation stages [48]; OCTN1 is highly induced (4-fold higher RNA levels) during lactation in human mammary epithelial cells, thus being potentially responsible for drug accumulation in milk [48].

## 3. Functional and Structural Aspects of OCTN1

To better understand the roles of the SLC22 transporters in cell physiology, the SLC22 subclades have been analyzed by expanding the investigation beyond phylogenetic relationships [19], using data from GWAS and from in vivo and in vitro models. This comprehensive approach led to the identification of a wider functional subgroup including OCTN1 and OCTN2, together with the Slc22a21 gene coding for OCTN3, which has been lost in humans, and other transporters now considered as OCTN-related. These last transporters belong to the same SLC22 family and are SLC22A15 and SLC22A16, also known as FLIPT1 and FLIPT2. They share substrate specificity with the original OCTN subgroup. This novel approach also highlights the limitations of using a single experimental approach to gain information on the role of transporters. 

### 3.1. OCTN1 Substrate Specificity and Relationships with Human Physiology

It should be stressed that in vivo measurements performed in rodents cannot be directly transferred to humans because of species differences in protein functions, expression, membrane location, regulation, and substrate specificity between rodents and humans [7,49], as it was also reported for other transporters of the same family [50] or of other families [51]. Thus, clarifying substrate specificity of the human protein is mandatory for understanding the role of human transporters in metabolism. Unequivocal answers to this issue are still lacking since many compounds have been identified or suggested as possible substrates of OCTN1. Intriguingly, the many suggested substrates, reported in Table 1 in alphabetical order and in Figure 1, include mainly cations and zwitterions belonging to different classes of molecules (see Tables for references).

Discrepancies are also reported on identification of OCTN1 substrates, which could be due to the mode of detecting transport in different experimental models. Mice, intact cells, and single protein assays (proteoliposomes) have been used as models. Moreover, different methodologies for transport monitoring have been employed, such as radiolabeled tracer assays, fluorescence measurements, liquid chromatography tandem mass spectrometry (LC-MS/MS), liquid chromatography triple quadrupole mass spectrometry (LC-TQMS), labelling reagent 3-aminopyridyl-Nhydroxysuccinimidyl carbamate (APDS) to derivatize OCTN1 substrates, etc. [10,11,12,13,14,16,18,28,52]. Some methodological limitations emerged, for example, in the case of LC-MS/MS in measuring the intracellular levels of cytarabine tested as an OCTN1 substrate [53]. This approach is not appropriate in the case of substrates that can undergo rapid enzyme-mediated metabolism inside cells. This problem is, at least partially, avoided by using radioactive isotopes to label substrates, which traces both the substrate and its metabolite(s).

An inhibition test on acetylcholine or other compounds (acetylcarnitine, choline, carnitine, metformin, pyrilamine, gabapentin, quinidine, verapamil, tetraethylammonium, neostigmine and the ergothioneine metabolites hercinine and S-methylergothioneine) was described in wild type HeLa cells, which express OCTN1, as well as in OCTN1 transfected HEK293 cells. In these models, ergothioneine transport was monitored by liquid chromatography mass spectrometry in the absence or in the presence of the compounds. Authors described a lack of inhibitory effect by any of the tested compounds on ergothioneine transport [54]. These findings questioned the role of all these molecules as OCTN1 substrates/inhibitors. However, in other experiments performed in the same cell system, i.e., HeLa cells [12], acetylcholine has been directly characterized as a low affinity substrate according to the role of OCTN1 in mediating a slow cell efflux that may play a role in the non-neuronal cholinergic system [11,55]. Moreover, ergothioneine was described as a very poor inhibitor of acetylcholine transport in proteoliposomes [54] reconstituted with the recombinant human OCTN1 or with the rat peritoneum OCTN1 [11,13]. This data correlates with the lack of inhibition by acetylcholine on ergothioneine transport, suggesting different transport paths.

### 3.2. Structure/Function Relationship

The divergent data on substrate specificity described could be explained by the existence of more than one binding site for the substrate(s) that could justify the lack of reciprocal inhibition by acetylcholine and ergothioneine (or other substrates). Based on this hypothesis, OCTN1 structure would contain two different or partially overlapping sites that could accept different classes of molecules. Each site should be characterized by amino acid residues conferring the specificity for different chemical scaffolds [6,9]. A similar feature was described in details for the OCTs [56]; three binding sites per transporter monomer of the rat OCT1 were identified [57]. Two low-affinity sites would be directly engaged in transport and the third site would exert allosteric control on the low-affinity sites [57]. Interestingly, the high-affinity site was only accessible to substrate in specific lipid environments. This finding highlights the involvement of the membrane lipid composition in regulating substrate specificity [35] and strengthens the influence of lipid–protein interactions on the membrane embedded transporter. Another example of a lipid–protein interaction in the SLC22 family is that of the carnitine transporter OCTN2 for which the influence of cholesterol on the substrate affinity has been described in HEK293 cells over-expressing the transporter. Interestingly, in this case, parallel experiments performed in the proteoliposome model corroborated the data obtained in intact cells [58]. Other papers report data concerning the cholesterol influence on OCTs [59,60,61]. Lipid regulation has been investigated for OCTN1, as well. In this case a more water soluble analogue of cholesterol has been used, which stimulated transport activity by increasing the Vmax of transport but not affecting Km, suggesting a direct interaction of the lipid with the protein [59]. It is also known that regulatory properties could physiologically change the ability of the transporter to recognize a given substrate based not just on membrane lipid composition but also on post translational modifications, protein–protein interactions and oligomerization, as described for OCT1 and OCT2 [60,62]. However, further investigations are needed to assess possible roles of lipid–protein interactions or post-translational modifications on OCTN1 specificity regulation. 

**Figure 2:**
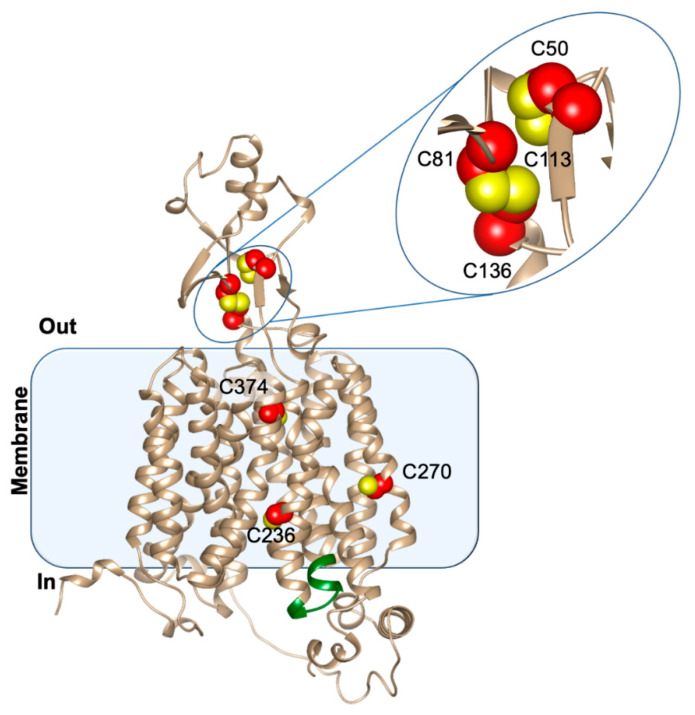
Ribbon representation of the OCTN1 structural model retrieved from Alphafold database. Cysteine residues are depicted as red spheres with Sulphur atoms in yellow. The intracellular nucleotide binding site is colored in green.

Some data obtained by using homology modeling approaches highlight that the cationic substrates as well as the zwitterion ergothioneine could interact with residues located in the transmembrane core of the protein [6]. Besides the major hydrophobic membrane domain, OCTN1, as well as the other members of the SLC22 family, the OCTs, possesses unusually large hydrophilic loops that could take part either in substrate binding or in regulation. The sole homology model containing a predicted structure of the large hydrophilic loop is available at the alphafold web site (https://alphafold.ebi.ac.uk (accessed on 12 November 2021) (see Figure 2). A close-up view of cysteine 50, 81, 113 and 136 is shown in Figure 2, which are located in the large extracellular loop and may be involved in the formation of disulfides, as also predicted on the basis of previous site-mutagenesis analysis [61]. This could indicate a potential redox control of the transporter’s structure/function in agreement with the suggested OCTN1 role in oxidative stress. Information on the transport mode of OCTN1 derives from different experimental models and approaches [10,11,63,64]. The various studies concur that the transporter mediates a sodium dependent or independent unidirectional transport depending on the transported substrates. Ergothioneine transport is sodium dependent, whereas acetylcholine transport is inhibited by sodium. An organic cation/proton antiport was also proposed [6,15,40].

## 4. Interaction with Endogenous/Exogenous Substrates

Two main features of transport specificity that have been confirmed by different works in which different methodologies have been used are the ability of OCTN1 to transport TEA and the scarce efficiency in transporting carnitine. Substitutions of specific amino acids of OCTN1 by a limited number of point mutations has been performed and several mutants allowed a 35% improvement in carnitine transport [52]. Interestingly, the zwitterionic substrate ergothioneine and the cationic substrate acetylcholine, which share some structural features with carnitine (Figure 1a), are also transported. Other substrates have been characterized by one or more methodologies/experimental models and are still being analyzed. Indeed, among the many proposed OCTN1 substrates, the most widely studied, besides TEA, which is a prototype substrate, are carnitine, ergothioneine and acetylcholine.

### 4.1. OCTN1 Substrate Specificity

#### 4.1.1. Carnitine

In the experimental systems described by most authors, carnitine is found to be a weak substrate. Although this is a widely recognized assumption, recent studies reported the role of OCTN1 in carnitine transport. In the mouse Motor Neuron-Like NSC-34 cell line, carnitine transport has been reported to be associated with both OCTN1 and OCTN2 [65]. In addition, impairment in carnitine transport by a broad range of antiretroviral drugs has been described in the placenta. Indeed, antiretroviral therapy during pregnancy could have adverse effects in newborns due to impairment of carnitine absorption. The authors, using fresh villous fragments, evaluated the OCTN1 and OCTN2 involvement in carnitine transport. This study confirmed the predominant expression and role of OCTN2 in L-carnitine transport [8]. Notwithstanding, [^14^C]-carnitine transport was adopted to investigate OCTN1 as a drug transporter [66]. In other reports, OCTN1 has been associated with fat metabolism. In two Iranian sheep breeds differing in tail phenotype (fat vs. thin), an OCTN1 gene variant was found to be involved in shaping the fat-tail, very probably stimulating fatty acid oxidation [67]. This last finding, however, is not necessarily correlated to carnitine. Low-efficiency transport of carnitine has also been revealed by our research group in proteoliposomes harboring recombinant human OCTN1, similarly to findings described by other groups using different experimental models [16]. Overall, it can be assessed that carnitine is accepted by OCTN1 to some extent and it cannot be excluded that in specific/different experimental conditions, such as presence of ions or OCTN1 interactors, the carnitine transport might increase.

#### 4.1.2. Ergothioneine

**Figure 3:**
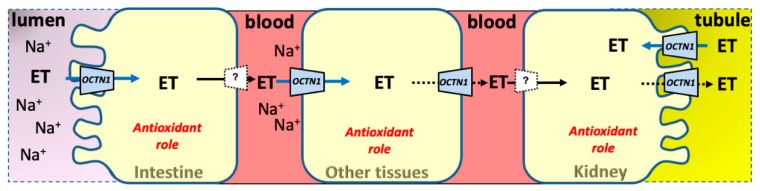
Metabolism and cellular role of the OCTN1 hypothesized substrate Ergothioneine ET. Epithelial polarized and other tissues are depicted as brushed or normal shapes. Intestine on the left, kidney on the right; other tissues in the middle. Intestine and tubular lumens are depicted in pink and yellow, respectively. Blood is depicted in red. Continuous arrows describe the OCTN1 transport of Ergothioneine, dotted arrows refer to a very low transport rate. Dotted lines represent other transporters than OCTN1. Transport regulation by sodium and adenosine triphosphate is reported.

Ergothioneine is a not physiological substrate. It is produced by fungi and actinobacteria but not by plants and animals, thus mushrooms are considered the major source for humans. It is present in human blood after diet absorption [68]. A study indicated that *Lactobacillus reuteri* may produce ergothioneine in vitro, even though no definitive evidence shows that gut microbiota can produce it; the presence of ergothioneine in a microorganism could be due to uptake from the environment and not to metabolic production [63]. In any case, the microbiota could interfere with host ergothioneine absorption. Ergothioneine has antioxidant properties (Figure 3). It cannot be considered a vitamin since it is not required for human metabolism. Indeed, a decline of ergothioneine in the human body is not associated with any disease [36]. Even though a higher sensitivity to oxidative stress in octn1 knockout mice with respect to wild type mice has been observed, any health benefits of ergothioneine have not been definitively assessed yet [42]. Some findings suggest that ergothioneine’s function as an antioxidant only becomes relevant following chronic exposure to oxidative stress, i.e., following depletion of other antioxidants [63]. A hypothesis is that OCTN1 expression would increase in damaged tissues and ergothioneine accumulation would serve to protect against further damage, suggesting that the OCTN1–ergothioneine axis may represent an adaptive antioxidant system [64]. Considering OCTN1 expression in rat placenta and human mammary glands, potential benefits arising from ergothioneine absorption have been hypothesized in offspring but not confirmed so far [22,69,70].

Ergothioneine is only partially involved in the regulation of neuronal differentiation, neuronal maturation, and microglial activation in vitro [18,71].

Indeed, in the brain and in the blood–brain barrier, a higher expression level has been found for another ergothioneine transporter, SLC22A15, involved in transport in this district, even though at a much lower efficiency [72]. Ultimately, although ergothioneine does not appear to play an essential role in human metabolism, some correlations have been found among its presence, OCTN1 expression, and anti-inflammatory effects [63].

#### 4.1.3. Acetylcholine

**Figure 4:**
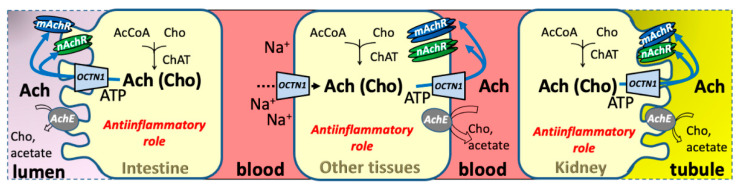
Metabolism and cellular role of the OCTN1 hypothesized substrate Acetylcholine, Ach (Choline, Cho). Epithelial polarized and other tissues are depicted as brushed or normal shapes. Intestine on the left, kidney on the right; other tissues in the middle. Intestine and tubular lumens are depicted in pink and yellow, respectively. Blood is depicted in red. Continuous arrows describe the OCTN1 transport of Acetylcholine (Choline); dotted arrows refer to a very low transport rate. Simplified human metabolic pathways are depicted: acetylcholine synthesis, involving the enzyme choline acetyltransferase (ChAT), and acetylcholine catabolism involving the enzyme acetylcholinesterase (AchE; gray circle); receptor interactions are reported (nAchR and mAchR, green and blue barrel shapes, respectively). Transport regulation by sodium and adenosine triphosphate is shown.

Acetylcholine transport mediated by OCTN1 has been demonstrated both in proteoliposomes and in intact immortalized or primary cell systems. Acetylcholine transport has been measured as radiolabeled acetylcholine accumulation in HeLa and mesothelial cells, which express endogenous OCTN1 [12,13]. Proteoliposomes reconstituted with membrane extracts from these cell lines also show acetylcholine transport activity similar to that of the recombinant human OCTN1 [11,12,13]. The transporter should play the major role of mediating acetylcholine export. Indeed, OCTN1 mediated uptake is inhibited by extracellular sodium, whereas the efflux can occur under physiological conditions. Based on the ability of OCTN1 to mediate acetylcholine export [11], a suggested role for OCTN1 is contributing to the cholinergic anti-inflammatory pathway, also in non-neuronal tissues, where the transporter is expressed. Indeed, acetylcholine can be synthesized in non-neuronal cells as well, due to the expression of choline acetyltransferase, as it has been demonstrated in several tissues, also in primary mesothelial cells [13,55]. Recently, the last International Symposium on Non-Neuronal Acetylcholine clarified that acetylcholine exported from cells plays biological effects resulting from interactions on two receptor subtypes mAChRs and nAChRs simultaneously stimulated and/or cross-talking among each other (Figure 4) [73]. This would contribute to the continuous cellular adaptation to body signals or to environmental conditions. This hypothesis correlates well with a possible role of OCTN1 in acetylcholine cellular export, thus contributing to healthly body functioning. Regulation of the acetylcholine transport by ions and ATP has been demonstrated for the native and the recombinant OCTN1 [11,12,63]. Transport of acetylcholine mediated by OCTN1 is stimulated by cholesteryl hemi succinate [59]. The stimulation occurs by a direct interaction of the cholesterol derivative with the protein, increasing the intrinsic transport function.

The ability to transport acetylcholine makes OCTN1 expression relevant also in the nervous system where the substrate acetylcholine (or choline) could enter through other transporters (SLC22A15) or alternatively could be produced in situ [12,13,20,74].

### 4.2. Knocking Out OCTN1 to Understand Substrate Selectivity

In the case of OCTN1 knockout obtained by disrupting mouse octn1 gene by homologous recombination [69], a deficiency of ergothioneine among 112 metabolites examined through metabolome analysis was found. However, the knockout mouse was characterized by lack of any apparent phenotype. Most probably, ergothioneine, which is an exogenous compound, is not the sole OCTN1 substrate and, after knocking it out, other transporters that share specificity for substrates with OCTN1 compensate for *OCTN1* gene deletion [7]. It could be that effects of knocking it out become evident only under specific conditions of metabolic stress [32,75,76]. To test this hypothesis, a gut ischemia and reperfusion condition was generated in mice mimicking a small-intestine inflammatory model. Indeed, after reperfusion, lethality was significantly higher in the octn1^−/−^ mice than in wild-type mice [69]. Histology of the intestinal mucosa showed loss of villus structures in the knockout. The octn1 knockout model has also been used in mice affected by Chronic Kidney Disease (CKD) [42]. In this pathological context the absence of octn1 in the knockout mice exacerbated renal fibrosis. However, the contribution of the deletion of renal octn1 to kidney injury in CKD mice remains unclear and cannot be ascribed to the sole defect of the antioxidant ergothioneine [42]. A Diabetes induced Kidney Disease (DKD) model was realized in knockout or wild type mice [70]. In knockout mice, the levels of a representative molecule of oxidative stress, the urinary 8-hydroxy-2′-deoxyguanosine (8-OHdG) were increased and interstitial fibrosis progressed more than in diabetic wild type mice [70,77] (see Section 6).

Furthermore, in octn1 knockout mouse, changes in the effects of drugs were also described. The biguanide metformin has been found to reach higher plasma concentration in octn1 knockout mice with respect to wild-type ones after low dose administration. On the contrary, lower plasma concentration levels of the drug were found in octn1 knockout after high dose administration [30]. After intravenous administration, elimination of metformin was similar in the two strains. This complex behavior suggests a possible involvement of OCTN1 in the gastrointestinal metformin transport at least at high doses. At lower dose, the transporter might be involved in intestinal metformin efflux, not uptake. In contrast, in the kidney, OCTN1 does not seem to be implicated in metformin excretion, considering that minimal differences in renal distribution between wild-type and knockout mice were observed [30]. 

## 5. Interaction with Drugs

Studying drug transport through the cell membrane is now a mandatory practice preceding preclinical pharmacodynamics and pharmacokinetic studies. The latter are mostly performed in rats or mice. Hence, rat kidney slices represent a useful system for evaluation of renal drug disposition via the expressed apical transporters like OCTN1 [78]. The kidney is one of the districts with relevance to pharmacokinetics and pharmacodynamics studies together with the intestine and liver. Renal secretion of organic cations mainly occurs in renal proximal tubules where OCTN1 has been confirmed to be apically expressed and where it would contribute to cation secretion [21]. The choice of the experimental system for investigating drug transport depends on the pharmacokinetic step object of study. In the case of the intestine, OCTN1 is included among transporters potentially involved in intestinal drug absorption and the human Caco-2 cells represent an experimental system of choice in studying the molecular process responsible for drug absorption.

An immortalized cell line was employed to study the xenobiotic transporters found at the blood–testis barrier potentially involved in drug disposition. To this aim, a human telomerase reverse transcriptase-immortalized human SC line (hT-SerC) was generated starting from the primary human Sertoli Cell (SerCs), compared to which it exhibited similar phenotypic characteristics [49] overcoming some of the disadvantages associated with the use of primary cells. Despite the advantages associated with this system, important limitations emerged from the analysis of mRNA expression for common xenobiotic transporters in immortalized hT-SerCs compared to hSerCs; in fact, even though a negligible difference in most cases (the expression remained within the 2-fold change threshold) was detected, in RT-qPCR and microarray a robust up-regulation of OCTN1 (by 2.3-fold and 3.5-fold, respectively) was found [49].

Involvement of OCTN1 in drug transport has been repeatedly reported in many reviews, some of which are very recent [6,7]. As in the case of physiological substrates, various conflicting data have been published on drug transport mediated by OCTN1, but unequivocal transport of certain drugs is now also recognized (see Table 2 and Figure 1b).

### 5.1. Controversial Data on Antineoplastic Drug Transport

Recently, the role of OCTN1 in mediating transport of the antineoplastic agent Cytarabine (Ara-C), a drug largely used in the therapy of Acute Myeloid Leukemia (AML), has been the object of controversial findings. In an in vitro system, a contribution by ENT1 and ENT2 in Ara-C uptake was demonstrated. In a later study, mice knocked out for ENT1 and for other putative Ara-C transporters including OCTN1 have been used [53]. Differently from what was observed in vitro, the pharmacokinetic properties were not altered in ENT1-knockout mice. OCTN1 deficiency does not influence the levels of Ara-C in either plasma or whole blood. The existence of interspecies differences must be considered. Indeed, in HEK293 cells over-expressing human OCTN1, the Ara-C intracellular levels were relatively high. Moreover, this study has been useful in elucidating how critical the experimental system and the technique used for measuring substrate or drug accumulation are; the radioisotope methodology is the most reliable one. Indeed, the same experiment performed in HEK293 gave opposite results if Ara-C intracellular levels were measured by LC-MS/MS [53]. Moreover, altered OCTN1 expression would seem strictly linked to a decreased Ara-C intracellular transport in AML cell lines and, as a consequence, to a lower response to treatment; thus, evaluating the OCTN1 expression level would be useful in predicting patient’s survival [17]. Increased *OCTN1* promoter methylation has been found to be the molecular basis of decreased OCTN1 expression. Thus, variable pharmacodynamics responses may be related to variable inter-individual promoter methylation status. This finding allows *OCTN1* to be included in the list of the transporters epigenetically regulated [79]. Interestingly, novel nucleoside analogues show strong efficacy against myeloid leukemia, thus investigating the involvement of OCTN1 in their intracellular accumulation is very important [80]. Other controversial data on the involvement of OCTN1 in drug transport concern another drug representing the best treatment option for chronic myeloid leukemia, the cationic tyrosine kinase inhibitor, imatinib [74]. Drugs belonging to this class are currently also used in a number of pathologies such as the rheumatic disease systemic sclerosis. Considering that OCTN1 is expressed in dermal fibroblasts, both healthy- and systemic sclerosis-derived, a therapeutic role in systemic sclerosis has been hypothesized for this transporter. Inhibition experiments performed with ergothioneine, however, demonstrated that OCTN1 activity would not have a significant role on the imatinib accumulation rate [75]. In order to draw correct conclusions, the used methodology should be considered once again and, as in the case of OCT1 [57], the presence of more than one substrate binding site has been postulated for OCTN1 as well, each involved in the recognition of specific substrates [6]. However, in only one study performed measuring radiolabeled imatinib accumulation in HEK293, no significant contribution by OCTN1 was found [76]; thus, further investigation is required.

For many other drugs, interaction/transport has been demonstrated and reported in prestigious reviews [6,7,15]; nevertheless, transport of certain drugs including nucleoside analogues, saracatinib, ipratropium, metformin and oxaliplatin has recently been challenged and only gabapentin uptake could be detected in HEK293 cells expressing human or rat OCTN1 [77].

### 5.2. Specificity of OCTN1–Drug Interactions

Specificity of the interaction between OCTN1 and drugs, as for substrates, has been proposed in some instances; a large series of drugs tested as substrates of OCTN1 based on their cationic nature or tested to investigate the molecular moieties responsible for the various steps of their pharmacokinetics, are neither transported nor interact with OCTN1. Some examples are reported here. Caco2 cells were used to assay the effect of OCTN1 inhibition on the cellular uptake of the eight main components from dragon’s blood phenolic extracts. No effect was found [81]. Based on the OCTN1 kidney localization and considering that the drug enalaprilat is excreted into the urine, OCTN1 has been tested together with other kidney transporters to identify the molecular entity responsible for enalaprilat excretion. To this aim, HEK293 cells overexpressing OCTN1 were used, and transport activity was evaluated following [^14^C]-carnitine transport in the presence of enalaprilat. Experimental data showed that OCTN1 is not involved in enalaprilat excretion [66], even though the use of carnitine as the transport tracer for OCTN1 may not be appropriated. The natural organic cation paeonol, exerting potential therapeutic effects against retinopathy, has been tested as a potential OCTN1 substrate. Indeed, OCTN1 is known to regulate the permeability of the Inner Blood–Retinal Barrier (iBRB). The involvement of OCTN1 in paeonol transport to the retina across the iBRB has been investigated following the uptake of [^3^H] paeonol by the conditionally immortalized rat retina capillary endothelial cells (TR-iBRB cell lines) used as an in vitro model of the iBRB. OCTN1 failed to transport paeonol [82]. Involvement of OCTN1 in absorption of naloxone and naltrexone, used to reverse opioid overdose, has been investigated. These opioid antagonists are absorbed by the human nasal epithelium containing appreciable levels of OCTN1, but negative results have been described [83].

Transport of the cationic (at physiological pH) psychotropic drug amisulpiride has been ascertained in human (hCMEC/D3) and mouse (bEnd.3) brain endothelial cell lines (blood–brain barrier), in which OCTN1 expression was confirmed. A transporter inhibition assay was performed by incubating cells with [^3^H] amisulpride and the potential inhibitor ergothioneine. Amisulpride accumulation in hCMEC/D3 and b.End3 was unaffected by the presence of ergothioneine. On the basis of these results, the authors concluded that amisulpride could not be described as a substrate for OCTN1 [84]. This result is in contrast to previous findings in which amisulpiride transport was measured in HEK293 cells over-expressing hOCTN1 and the drug was quantified at the end of transport by HPLC [23]. In these experiments, transport was inhibitable by carnitine. Again, it would be appropriate to consider the methodology used before drawing conclusions.

OCTN1 has been investigated among the potential transporters responsible for heart accumulation of the natural compound dehydrocorydaline. Experiments performed in transgenic cells, primary neonatal rat cardiomyocytes, and animal experiments elucidated the involvement in the dehydrocorydaline transport of the OCT1 and 3 but not of OCTN1 [85]. Another example is given by the aconitum alkaloids, therapeutic agents used in the context of the traditional Chinese medicine; these alkaloids are responsible for neurotoxicity, which is a known important side effect. In this study, silencing OCTN1 by specific siRNA did not interfere with the uptake process of these drugs [86]. In a recent study, uptake of [^99m^Tc] dimercaptosuccinic acid ([^99m^Tc] DMSA) in renal tubular epithelial cells was investigated; this molecule highly accumulates in the kidney cortex and for this reason is a major renal cortical imaging agent used in the diagnosis of renal parenchymal disorders. Uptake experiments in HEK293 cells expressing OCTN1 revealed it was not responsible for [^99m^Tc] DMSA transport [87]. The antiepileptic lamotrigine provides a further example [88]. Recently, particular attention has been paid to the involvement of OCTN1 in the delivery of drugs through specific barriers such as the placenta, giving rise to a potential key role of OCTN1 in the transfer of drugs to the fetus. Indeed, OCTN1 is among the transporter objects of studies to investigate fetal drug disposition [47]. The aim of this study was to elucidate the transport mechanisms at the basis of the passage of lamotrigine in the placenta, considering that it is used to reduce seizures in pregnant women; to this aim BeWo and JEG-3 cell lines were employed but, in this case, OCTN1 was also not involved in placenta drug transport. Along with the BeWo, the primary human trophoblast cells (PHTCs) have been used as cell models of the placenta for investigating emtricitabine (FTC) accumulation [25]. This is a first-line antiviral drug recommended for the treatment of AIDS during pregnancy. The described findings indicated a possible contribution of OCTN1 in drug uptake from the maternal circulation to trophoblasts. However, the high apparent Km for the drugs together with the relatively low level of OCTN1 mRNA revealed in the human placenta, have suggested a possible major role of OCTN1 in FTC transport in the placenta. 

### 5.3. Toxicokinetics and Drug–Drug Interactions (DDI)

Owing to the potential involvement of OCTN1 in drug delivery to the fetus, a potential role in infants has been proposed as well. OCTN1 expression has been described in the mammary gland where OCTN1 would be involved, during lactation, in transferring nutrients and compounds of various types into milk, contributing to its composition. Considering the ability of the transporter to recognize drugs, their transfer into milk should be expected. A highly induced (4-fold higher RNA levels) OCTN1 expression found during lactation in human mammary epithelial cells could correlate well with this role [48]. 

Acting like an uptake transporter, OCTN1 would lead to an accumulation of drugs in tissues like the kidney, at the level of proximal tubular cells, potentially causing renal damage [22]. An example is given by cephaloridine, which together with other drugs is suggested to be a substrate of OCTN1 at the kidney level. 

Among the known OCTN1 transported drugs is oxaliplatin, a drug used to treat colorectal cancer and known for drug-induced neurotoxicity, in particular, peripheral neuropathy. Oxaliplatin accumulates in the dorsal root ganglion where OCTN1 was identified with real time PCR. Knockdown of OCTN1 ameliorated peripheral neuropathy, decreasing platinum accumulation [89]. Toxicokinetics could be influenced by Drug–Drug Interactions (DDI). In a recent study, L-tetrahydropalmatine has been found to attenuate peripheral neurotoxicity induced by oxaliplatin via inhibiting its transport but without affecting the antitumour efficacy of the drug [90]. Moreover, it is known that amisulpiride, cytarabine, entecavir, metformin, pregabalin, gapapentin, 5-aminosalicylic acid, and camptothecin are drugs whose interaction with OCTN1 is influenced by sulpiride [22]. 

Concerning drug–drug interactions, the interaction between gabapentin, a drug used to treat chronic pain, and the antiallergic cetirizine has been investigated in HEK293 cells overexpressing OCTN1 and a clinical trial was conducted in patients with neuropathic pain to evaluate the effect of cetirizine on gabapentin pharmacokinetics [91]. The clearance of the OCTN1 substrate gabapentin occurs at the kidney tubular level. Cetirizine is excreted at the kidney level as well. The hypothesis was that cetirizine would inhibit the renal secretion of gabapentin in kidney proximal tubules and therefore would increase gabapentin plasma concentration. Surprisingly, renal clearance did not change when drug co-administration varied by plasma gabapentin concentrations and pain attenuation, which were lower after co-administration. Data obtained suggest the involvement of the intestinal OCTN1 in this DDI [91]. Gabapentin pharmacokinetics were also evaluated in humans with diabetes and hyperglycemia. These pathological conditions do not interfere with gabapentin pharmacokinetics [92].

### 5.4. OCTN1 Polymorphisms and Drug Transport

Considering that *OCTN1* is a polymorphic gene, an important issue concerns the effect of polymorphisms on drug transport/disposition. Indeed, polymorphisms may be key determinants of variable pharmacokinetics and clinical response in individuals, increasing or decreasing the transporter activity and so determining inter-patient variability in drug response; thus, evaluation of the polymorphic variants could be useful as a predictor of time to progression. In a previous study, the c.1507C>T polymorphism, corresponding to the substitution of leucine 503 with phenylalanine (L503F-OCTN1), was associated with a decrease in active tubular secretion of gabapentin [28]. Most recently, it has been demonstrated that the genetic polymorphism c.1507C>T does not have a significant influence on gabapentin absorption, distribution or elimination; indeed, the pharmacokinetics would be influenced by renal function and absorption saturation processes [93]. Moreover, the *OCTN1*-917C>T (I306T-OCTN1) variant transports gabapentin less efficiently than WT-OCTN1 [24]. Other authors disagree with this finding, hypothesizing a role at the kidney level in drug secretion into the collecting duct [35]. The variants c.1507C>T and *OCTN1*-917C>T, both characterized by an amino acid substitution, have been reported to alter the pharmacokinetics of metformin; the former transports metformin more efficiently than WT-OCTN1; the second variant affected the apparent oral clearance (CL/F) of metformin calculated as the dose of metformin divided by AUC inf, where F is the oral bioavailability [94]. This finding suggests a possible role of OCTN1 in the secretion of metformin into the collecting duct [35]. In a further study the pharmacogenetics of imatinib has been studied in different sample sets, the most representative being that of Caucasians. In this study, the *OCTN1* C allele (rs1050152) corresponding to *OCTN1* WT (1507 C) was significantly associated with the major molecular response to this pathology [74]. Imatinib efficacy against chronic myeloid leukemia has also been investigated in Chinese Patients [95]. A panel of polymorphisms in the *OCTN1* gene was selected; in particular, three *OCTN1* variants were chosen for genotyping: 917 T>C, −248 C>G and −538 C>G. In all SNPs, there were no statistically significant relationship among imatinib concentrations and each SLC22A4 genotype [95]. In evaluating the impact of genetic polymorphisms on drug pharmacokinetics and responses, the inflammatory status of patients must be considered, which contributes to intra- and interindividual variability of drug exposure, modulating important Drug-Metabolizing Enzymes and Transporters (DMETs) [96].

Altogether, the reported findings highlight a well assessed involvement of OCTN1 in drug interactions. However, further efforts are required to definitively clarify the role and the specificity of OCTN1 in drug disposition.

### 5.5. Influence of Xenobiotics and Natural Compounds on OCTN1 Expression

The effects of single and repetitive valproic acid administration in pregnant rats on the expression of placental transporters and thus OCTN1 have been evaluated. As opposed to OCTN2, which showed no significant change, OCTN1 expression was influenced: it increased markedly following gestational development and decreased with valproic acid administration at the G20 stage [97].

Anthocyanins, novel xanthine oxidase inhibitors, are used to treat hyperuricemia. These compounds regulate cytokine expression, which in turn could modulate OCTN1 expression. Anthocyanins could rescue OCTN1 expression, which is down regulated in mice with hyperuricemia, improving renal function recovery. The same effect could be exerted by allopurinol [98]. In pathologies like hyperuricemia, OCTN1 could mediate anti-inflammatory effects through the action of the transported substrates (acetylcholine and/or ergothioneine) exerting a nephroprotective effect.

Circulating corticosteroids may influence expression level as well: they, and thus signals from the adrenal gland, influence rat intestinal expression of OCTN1 and are necessary for maintaining an OCTN1 upregulated expression. Thus, stress induced changes in the secretion of corticosteroids might reorganize OCTN1 intestinal expression [99]. 

*Cynara Cardunculus*, a component of the Mediterranean diet displaying hepato-protective properties, restored OCTN1 expression after impairment by a fat diet in the liver. The beneficial effect of *Cynara Cardunculus* seems to involve carnitine transport mediated by OCTN2 and OCTN1 [100].

## 6. Relevance to Human Pathology

The association between OCTN1 and inflammatory diseases is well established; in particular, starting with Peltekova’s et al. authoritative work [39], an association between the TC haplotype of *OCTN* consisting of the genetic variant responsible for the substitution of one amino acid (L503F) and susceptibility to Crohn’s disease has been validated by several other association studies [101]. The same variant has been associated with sporadic colorectal cancer in early age and in ulcerative colitis (UC) patients as well, linking severity of inflammation to cancer progression [102]. More recently, in a case report of Irritable Bowel Syndrome (IBS) characterized by a dysfunctional microbiota–gut–brain axis, involvement of the same variant previously associated with Crohn’s disease was found [103].

A role of OCTN1 in cancer has been reported [104]. This relationship has been further explored since, as an acetylcholine transporter, it is considered a component of the non-neuronal cholinergic system (see Section 4.1.3); as such, it should be involved in the pathophysiology of lung cancer [105]. Indeed, acetylcholine acts via nicotinic or muscarinic receptors modulating proliferation, induction of Epithelial–Mesenchymal Transition (EMT), migration and invasion of human lung cancer cells, etc. Thus, the role of OCTN1 in this context is strictly linked to that of receptors that could be expressed at a higher level in the early phases of tumorigenesis, declining with tumor progression to an advanced stage. Interestingly, functional nAChRs are not only localized on the outer cell plasma membrane but are also expressed on the outer mitochondrial membrane of lung tissues, which is in line with potential OCTN1 mitochondrial localization [106]. At this subcellular level, the receptors would have the pro-survival functions. However, to define a possible role of OCTN1 in mitochondria, its subcellular localization of OCTN1 needs to be definitively addressed, taking into account the possible mitochondria–plasma membrane interactions [107].

In a case report of pulmonary metastases from breast cancer, two types of lesions were genetically differentiated between drug-resistant and sensitive lesions. An OCTN1 variant was found in the sensitive lesion. This heterogeneity would suggest a different response to therapy among distant metastases in the same patient [108,109]. Indeed, the lesion associated with OCTN1 mutation was reduced by treatment with paclitaxel. 

These data suggest that OCTN1 is a possible drug target in cancer.

Recently, a population study elucidated the association among specific polymorphisms and tooth loss, the most prominent consequence of periodontitis [110]. 

An association of a variant with hearing impairment was found in Tunisian and Moroccan families as well [111,112]. The role of OCTN1 in normal hearing function has not been clarified considering that the octn1 knockout mouse lacks a phenotype, not showing apparent signs of hearing loss. 

A relationship between OCTN1 and epilepsy has been recently found [18]. Epileptic seizures were experimentally induced by repeated administration of pentylenetetrazole (PTZ) in octn1 knockout mice. Interestingly, a phenotype was observed: knockout mice showed much lower seizure scores compared with wild-type mice. Untargeted metabolomics analysis allowed identification of homostachydrine as responsible for deterioration of PTZ-induced seizures in the wild type brain. The authors suggest that ergothioneine, inhibiting homostachydrine uptake, decreases its concentration in the brain and thus, inhibits PTZ-induced kindling. Hence, OCTN1 could represent a suitable target for anti-epileptic drugs.

A relationship with oxidative stress has been suggested as well. In octn1 knockout and wild type mice, a diabetic kidney disease model was induced by spreptozotocin. In diabetic octn1 knockout mice, oxidative stress was higher and interstitial fibrosis progressed more rapidly than in diabetic WT mice. The authors speculated that oxidative stress was induced in the early phase (12–20 weeks) of the diabetic condition in octn1 knockout mice due to ergothioneine deficiency. Subsequently, some antioxidative processes, such as compensating specific gene expression, might be activated by reducing oxidative stress in octn1 knockout mice. Moreover, in diabetic kidney disease, octn1 might play a role in interstitial fibrosis progression by acting on expression of moesin, a protein that works as a cross-linker between the plasma membrane and actin-based cytoskeleton, and which plays a part in interstitial fibrosis even though the molecular mechanism is yet to be clarified. Moesin was up-regulated in octn1 knockout diabetic mice [70].

In chronic kidney disease, the role of OCTN1 in kidney–intestine cross-talk has been investigated and impairment of the OCTN1–ergothioneine axis has been proposed. This axis is considered as an adaptive antioxidant system because OCTN1 expression increases in damaged tissues and ergothioneine accumulates to protect against further damage. The ability of OCTN1 to absorb ergothioneine at the intestine level was diminished in mice with Chronic Kidney Disease (CKD) due to OCTN1 dysfunction. Indeed, OCTN1 localization on the intestinal apical cellular membrane was altered in mice with CKD due to a reduction in expression of PDZK1, an apical membrane scaffold protein [41,42]. These data suggested that the OCTN1–ergothioneine axis does not function in CKD because this pathological condition impairs intestinal OCTN1 activity and consequently decreases ergothioneine blood and body levels [42].

### 6.1. Pathologies Modulating OCTN1 Expression

Pathology can modulate the expression level of OCTN1 [7,16,42,113,114]. In Polycythemia Vera (PV) patients, OCTN1 expression has been found to be increased in hematopoietic stem/multipotent progenitor cells (HSC/MPPs) compared with common myeloid/megakaryocyte-erythrocyte progenitors (CMP/MEPs). OCTN1 is involved in the transport of hydroxyurea, a standard treatment for high-risk patients with PV. The findings suggest that a higher accumulation of hydroxyurea occurs in HSC/MPPs compared to CMP/MEPs, highlighting the importance of the knowledge of transporter expression in linking therapeutics with human disease [34]. 

In the context of muscular dystrophy, gene expression of mOctn1 mRNA in the quadriceps of adult mdx compared to wild-type mice was found to be increased [115].

OCTN1 expression in cancers has been reported as well [104], which is relevant considering OCTN1 as a route for antineoplastic agents (see Table 2). 

Inflammation can modulate transporter expression. OCTN1 down-regulation in inflammation could be mediated by cytokines. Data on the action of specific cytokines on OCTN1 expression have been reported in recent papers [116]. In turn, OCTN1 can regulate the induction of the inflammatory cytokine IL1-β via its substrate [117].

It has been demonstrated that simulated viral-induced acute inflammation activates cytokine-mediated pathways through the Toll-like receptor 3, inducing a reduction in kidney Octn1 mRNA levels at 6 but not 24 h in rats at mid-gestation. No information has been reported on protein expression level [118]. The same was found at term pregnancy at 24 h. Interestingly, Octn2 mRNA level was not affected [119]. In contrast, metabolic acidosis induced in rats caused an up-regulation in the kidney Octn1 at both the mRNA and protein level [120]. Another example is given by inflammation induced by LPS, which inhibits the expression and activity of OCTN1 in alveolar epithelial cells (A549), potentially reducing the distribution of inhaled medicine in pulmonary diseases [108]. LPS-induced inflammation down-regulated mammary gland OCTN1 mRNA expression at each lactation stage [121]. Cigarette Smoke Extract (CSE) combined with LPS was evaluated in the regulation of OCTN1 expression [113]. OCTN1 downregulation was detected both in rat lung in vivo and in a human alveolar cell line in vitro. In contrast, the inflammatory factors were up-regulated. Once again, the mechanism involved the Toll-like receptors (TLRs); endotoxin-binding protein (LBP) captures CSE or LPS, and then delivers it to the CD14 molecule to form a complex. The complex binds TLRs and activates Interleukin-1 Receptor Associated Kinase (IRAK). Finally, the signal is transferred to the inflammatory response related NF-κB signaling pathway. Further research is required to clarify the mechanism by which NF-κB regulates OCTN1. Interestingly, after treatment with ipratropium bromide (a nonselective muscarinic receptor inhibitor) or dexamethasone, the expression of OCTN1 was upregulated compared with that in the CSE-LPS model group [113]. Hence, the OCTN1 expression level is modified not just by physio-pathological conditions but also by xenobiotics and physiological and natural compounds.

## 7. Conclusions

In spite of discrepancies among the data collected so far concerning OCTN1 substrate specificity, it seems that all the disagreeing data converge to the same OCTN1 role. Indeed, results gathered so far allow us to conclude that similarly to OCTs, OCTN1 does not seem to be essential for life but most likely its physiological role has to do with better survival. This could explain the lack of phenotype in knockout mice and the appearance of a phenotype after stress induction. A recently described action of OCTN1 in conferring radioresistance [114] correlates with this hypothesis. Importantly, the involvement of OCTN1 in mediating cell release of the anti-inflammatory acetylcholine in non-neuronal tissues and in mediating absorption of the antioxidant ergothioneine are in favor of a role in better survival. 

In addition, the ability of OCTN1 to interact with molecules other than human metabolites suggests a possible role for this transporter in mediating communication between the human body and the external environment, e.g., the microbiota. In this frame, the transporter may realize an inter-organismal communication according to the remote sensing and signaling theory [122].

The ability of OCTN1 to transport or, at least, to interact with many drugs together with the described link of OCTN1 with several human pathologies, suggests that this transporter can be used for in vitro screening assays to predict drug delivery and toxicity at the molecular level. Therefore, OCTN1 should be considered for novel drug design and we expect it will be included in the FDA or EMA guidance soon. 

The hypotheses put forward so far by many research groups regarding the role of human OCTN1 in metabolism and in drug interactions will be better defined by the incoming studies. A significant boost is expected by the 3D structure solution that will unravel the molecular determinants responsible for specificity and for the molecular mechanism of transport. Clarification of the sub-cellular localization as well as the interaction with other proteins or regulators will also be important to complete the picture of the transporter, which certainly has a great, even though unclear, importance in human wellbeing.

## Figures and Tables

**Table 1:** Endogenous and Natural Substrates transported by OCTN1. For each substrate, Description/Role and one or more References are reported.

Transported Endogenous/Natural Substrates	Description/Role From Pubmed Compound Database	References
Acetylcholine	Acetate ester and an acylcholine. Human metabolite	[11]
Carnitine	Amino-acid betaine. Human metabolite	[15,16]
Choline	Parent compound of the cholines class. Human metabolite	[12]
2′-Deoxycytidine	Cytidine analogue. Human metabolite	[17]
Ergothioneine	L-histidine derivative. Fungal metabolite	[10]
Homostachydrine	Ammonium betaine. Plant metabolite	[18]
Spermine	Polyazaalkane. Antioxidant, immunosuppressive agent and human metabolite	[14]
Stachydrine	L-proline betaine. Plant metabolite	[10]

**Table 2:** Drugs transported by OCTN1. For each drug, Description/Role and one or more References are reported.

Transported Drugs	Description/Role From Pubmed Compound Database	References
Amisulpiride	Member of pyrrolidines. Antipsychotic/antischizophrenic agent	[23]
Buformin	Class of biguanides. Antidiabetic drug	[24]
Clofarabine	Adenosine analogue. Antineoplastic drug	[17]
Cytarabine	Cytidine analogue. Antiviral and antineoplastic drug	[17]
Emtricitabine	Nucleoside analogue. Antiviral drug	[25]
Entecavir	Nucleoside analogue. Antiviral drug	[26]
Ethambutol	Ethylenediamine derivative. Antitubercular drug	[27]
Fludarabine	Adenosine analogue. Antineoplastic drug	[17]
5-Fluorouracil	Pyrimidine analogue. Antineoplastic activity	[17]
Gabapentin	γ-amino acid. Anticonvulsivant, treatment of neuropathic pain	[24,28]
Gemcitabine	Cytidine analogue. Antineoplastic drug	[17]
Ipratropium	Quaternary ammonium ion. Anticholinergic drug	[29]
Metformin	Class of guanidines. Hypoglycemic drug	[24,30]
Oxaliplatin	Organoplatinum complex. Antineoplastic drug	[31]
Phenformin	Class of biguanides. Antidiabetic drug	[24]
Pyrilamine	Ethylenediamine derivative. H1-receptor antagonist	[15]
Quinidine	Cinchona alkaloid. Antiarrhythmic and antimalarial effects	[15]
Ribavirin	Guanosine analogue. Antiviral drug	[17]
Saracatinib	Class of quinazolines. Antitumor activity	[32]
Tea	Quaternary ammonium ion. Experimental drug	[15,16]
Tiotropium	Quaternary ammonium ion. Muscarinic antagonist and bronchodilator drug	[29]
Verapamil	Tertiary amino compound. Calcium channel blocker	[15]
